# Prediction of brain invasion in patients with meningiomas using preoperative magnetic resonance imaging

**DOI:** 10.18632/oncotarget.26313

**Published:** 2018-11-13

**Authors:** Alborz Adeli, Katharina Hess, Christian Mawrin, Eileen Maria Susanne Streckert, Walter Stummer, Werner Paulus, André Kemmling, Markus Holling, Walter Heindel, Rene Schmidt, Dorothee Cäcilia Spille, Peter B. Sporns, Benjamin Brokinkel

**Affiliations:** ^1^ Institute of Clinical Radiology, University of Münster, Münster, North Rhine-Westphalia, Germany; ^2^ Institute of Neuropathology, University Hospital Münster, Münster, North Rhine-Westphalia, Germany; ^3^ Institute of Neuropathology, Otto-von-Guericke University, Magdeburg, Saxony-Anhalt, Germany; ^4^ Department of Neurosurgery, University Hospital Münster, Münster, North Rhine-Westphalia, Germany; ^5^ Institute of Biostatistics and Clinical Research, University of Münster, Münster, North Rhine-Westphalia, Germany

**Keywords:** brain invasion, grading, meningioma, magnetic resonance imaging, radiology

## Abstract

Brain invasion (BI) in meningiomas impacts WHO grading and therefore adjuvant treatment. However, BI is rare and neurosurgical sampling and neuropathological analyses are not standardised. Moreover, associations with imaging findings are sparsely known. Associations between BI and findings on preoperative MRI were investigated in 617 meningioma patients. BI was strongly correlated with other high-grade criteria (p<.001). Presence of a contrast enhancing tumour capsule, disruption of the arachnoid layer, intratumoural calcifications and T2-intensity were not related to high-grade histology or BI (p>.05, each). High-grade histology (p=.033) but not BI (p=.354) was associated with tumour location. Irregular tumour shape (OR: 3.33, 95%CI 1.33-8.30; p=.007), heterogeneous contrast enhancement (OR: 2.82, 95%CI 1.19-6.70; p=.015) and peritumoural edema (OR: 1.005 per ccm, 95%CI 1.001-1.008); p=.011) were associated with BI. Multivariable analyses identified only increasing edema volume (OR: 1.005 per ccm, 95%CI 1.002-1.009; p=.010) as a predictor for BI, independent of other histopathological high-grade criteria. We finally provide a new model to estimate the risk of BI using routine preoperative MRI. Several imaging characteristics were identified as predictors for BI. Consideration in clinical routine can increase the accuracy of the detection in neuropathological analyses.

## INTRODUCTION

With the release of the 2016 edition of the WHO Classification of Central Nervous System Tumours, microscopical detection of brain invasion has been added as a stand-alone grading criterion in meningiomas [1]. Hence, detection of brain invasion in neuropathological analyses has gained distinct clinical relevance as directly impacting grading and therefore eventually decision making about adjuvant irradiation and trial inclusion [2–5]. Moreover, further studies suggest brain invasion as an important predictor for perioperative complications. Hence, brain invasion was correlated with preoperative behaviour changes [6]. Similarly, brain invasion was identified as a strong predictor of preoperative seizures independent of patients age, sex, WHO grade and, remarkably, of tumour location, peritumoral edema or tumour volume (OR 5.26, 95% CI 1.52-18.15; p=.009) [7]. In a recent study, the risk of postoperative haemorrhage was more than 3-fold increased in patients with brain-invasive as compared to individuals with non-invasive meningiomas [8].

While brain invasive growth is clearly defined in the WHO classification of brain tumours, both neurosurgical sampling and neuropathological analyses are not standardised yet [4, 5, 9]. Correspondingly, reported frequencies of brain invasion in neuropathological tissue samples distinctly vary [2] and a considerable portion of invasive meningiomas might not be detected during microscopical analyses. In line with this hypothesis, extensive and systematic sampling during neuropathological analyses were shown to increase the detection rates of brain invasion [10].

On the other hand, preoperative clinical or radiological variables associated with brain invasion are largely unknown. A few studies investigated associations between brain invasive growths and peritumoural brain edema (PTBE) with partially inconclusive results [2, 7, 10, 11]. Other series revealed associations between findings on preoperative MRI and high-grade histology without separately analyzing brain invasion as a stand-alone grading criterion [12, 13]. However, identification of associated risk factors could decisively help to improve the sensitivity of the detection of brain invasion in microscopic analyses and is therefore urgently needed.

In this series, we therefore investigated associations between brain invasive growth during microscopical analyses and findings assessable on routine preoperative magnetic resonance imaging (MRI).

## RESULTS

Using the above-described approach, 1104 patients who underwent surgery for intracranial meningioma were identified. 617 individuals with sufficient imaging in terms of preoperative T1-weighted contrast-enhanced MRI were included in this study comprising 176 males (29%) and 441 females (72%) with a median age of 59 years (range: 7-91 years). Surgery was performed for primary diagnosed meningiomas in 570 (92%) individuals but for recurrent tumours in 47 patients (8%). Table 1 summarises histopathological and radiological variables subjected to statistical analyses.

**Table 1:** Summarization of radiological and histopathological data of patients with primary diagnosed and recurrent meningioma

Variable	Available data (N, n%)	Frequency (N, n%)
**Tumour location**	617 (100%)	
Convexity		215 (35%)
Falx/parasagittal		85 (14%)
Skull base		271 (44%)
Posterior fossa		41 (7%)
Intraventricular		5 (1%)
**Tumour/edema volume**		
Tumour volume (median, range)	554 (90%)	12.71 ccm (0.02-356.94 ccm)
Edema volume (median, range)	529 (86%)	0.00 ccm (0.00-739.28 ccm)
**Intensity on T2-weighted MRI**	540 (88%)	
Hypointense		294 (48%)
Isointense		19 (3%)
Hyperintense		227 (37%)
**Further radiological criteria**		
Archnoid layer disrupted/absent	531 (86%)	296 (48%)
Heterogeneous T1 contrast enhancement	617 (100%)	262 (43%)
Tumour shape irregular	558 (90%)	225 (37%)
Tumour calcifications	554 (88%)	115 (19%)
Capsular contrast enhancement	523 (85%)	160 (26%)
**WHO Grade**	617 (100%)	
WHO grade I		557 (90%)
WHO grade II		57 (9%)
WHO grade III		3 (1%)
**Brain invasion**	617 (100%)	
Present		24 (4%)

The left column delineates the rate of available data, the right column shows the frequency of the corresponding variable and the median/range of the tumour and PTBE volumes.

### Histopathological data

Brain invasive growth was detected in of 24 of all 617 cases (4%) and was found in 23 of 57 atypical (40%) and 1 of 3 anaplastic meningiomas, while it was absent by definition in all 557 grade I lesions (p<.001). In atypical meningiomas, grading exclusively based on the microscopic evidence of brain invasion in 19 individuals (33%), while further histopathological criteria for atypia were lacking (“otherwise benign” lesions). In the remaining atypical meningiomas, grading solely based on other histopathological criteria of atypia in 34 patients (60%) or a combination of both in 4 individuals (7%). Detection of brain invasion during microscopic analyses was strongly correlated with the presence of further histological criteria of atypia or anaplasia both in the entire cohort (p<.017) and in subgroup analyses of high-grade meningiomas (p<.001). Brain invasion was found in 13 of 441 female but in 11 of 176 male meningioma patients (3% vs. 6%, p=.066).

### Associations of high-grade histology with findings on radiological imaging

**Figure 1:**
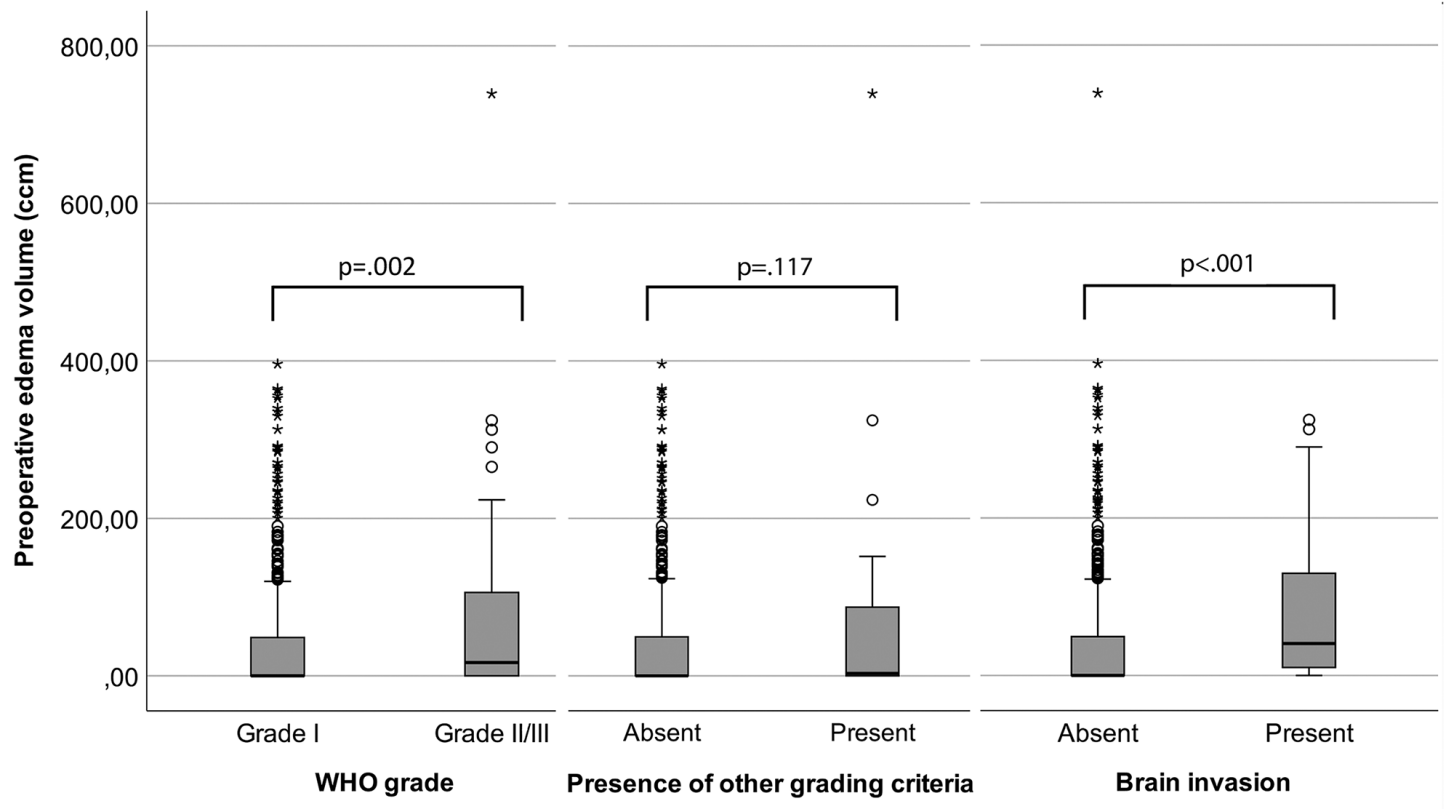
Boxpots visualizing the degree of association between peritumoural edema (PTBE) volume and histopathological findings High-grade histology was associated with increased PTBE volume (left, p=.002) and PTBE volumes were larger in invasive than in non-invasive meningiomas (p<.001, right). However, no association was found between edema volume and other histopathological grading criteria (p=.117). The boxes indicate upper and lower 25% quartile, the whiskers the minimum/maximum value within 1.5 IQR of the lower/upper quartile, the dots the outliers, the asterisks the extreme values, and the heavy horizontal line indicates the median (ccm=cubic centimeter, *high-grade=grade II and III meningiomas.).

In univariable analyses, no associations between tumour intensity on T2-weighted MRI (p=.084), disruption/absence of the arachnoid layer (p=.660), calcification (p=.727) or capsular contrast enhancement (p=0.635) and high-grade histology were found. However, high-grade histology was found relevantly more often in convexity or falx meningiomas than in tumours of other locations (16% vs. 8%, p=.033) and more often in tumours with irregular as compared to regular shape (56% vs 44%, p=.013). Moreover, 38 of 60 high-grade but 224 of 557 benign meningiomas displayed heterogeneous contrast enhancement (63% vs. 40%, p=.001). Median tumour (20.26 ccm, range: 1.00-172.90 ccm vs. 10.60 ccm, range: .02-356.94 ccm; p=.002) and PTBE volumes (17.00 ccm, range: .00-739.28 ccm vs .00 ccm, range: .00-364.63 ccm; p=.002, Figure 1) were higher in high-grade than in benign meningiomas.

### Association of high-grade criteria other than brain invasion with findings on radiological imaging

Subsequently, assocations between the presence of histopathological grading criteria other than brain invasion and findings on preoperative imaging were investigated. Similar to the above mentioned results, the presence of high-grade criteria other than brain invasion on microscopic analyses was not associated with tumour intensity on T2-weighted MRI (p=.109), disruption/absence of the arachnoid layer (p=.603), tumour shape (p=.124), PTBE volume (p=.117, Figure 1), calcification (p=.676) or capsular contrast enhancement (p=.266). Noticeably more non-invasive high-grade tumours harboring other criteria of atypia/anaplasia than benign meningiomas were located at the convexity or parasagittal (71% vs. 49%, p=.013). Heterogeneous contrast enhancement was found in 26 of 41 non-invasive but high-grade tumours but in only 236 of 576 benign meningiomas (63% vs. 41%, p=.008). Median tumour volume was 16.35 ccm (range: 1.00 ccm-172.90 ccm) in non-invasive high-grade meningiomas as compared to 11.18 ccm (range: .02-365.94 ccm) in their benign counterparts (p=.016).

### Brain invasion and findings on radiological imaging

Table 2 summarises associations of brain invasion with findings on preoperative imaging. In univariable analyses, no association was found between brain invasion and the tumour intensity on T2-weighted MRI images (p=.310), intratumoural calcifications (p=.808), capsular contrast enhancement (p=.372), tumour location (p=.354), tumour volume (p=.588) or arachnoid layer (p=.895). However, in univariable analyses, invasion of the adjacent brain was associated with increasing edema volume (OR: 1.005 per ccm; 95% CI 1.001-1.008; p=.011, Figure 1), irregular tumour shape (OR: 3.33, 95% CI 1.33-8.30; p=.007), and heterogeneous contrast-enhancement (OR: 2.82, 95% CI 1.19-6.70; p=.015). ROC analysis suggested a cut-off point for edema volume at 3.64 ccm as discrimination threshold for brain invasion. The AUC was 0.718 (95% CI 0.610-0.826).

**Table 2:** Association between brain invasion and clinical and radiological variables in uni- and multivariable logistic regression

Variable	Univariable analysis: OR (95% CI)	p-value		Multivariable analysis: OR (95% CI)	p-value	
Gender: Male vs female (ref.)	2.20 (0.96 to 5.00)		p=.067	2.45 (0.81 to 7.40)		p=.113
Age at surgery (in years)	1.022 (0.992 to 1.052)		p=.146	N/S		p=.149
Tumour location: Convexity/ falcine vs other (ref.)	1.57 (0.60 to 4.13)		p=.354	N/S		p=.490
Tumour volume (in ccm)	1.003 (0.993 to 1.013)		p=.588	N/S		p=.791
Edema volume	1.005 (1.001 to 1.008)		p=.011	1.005 (1.002 to 1.009)		p=.010
Intensity on T2-weighted MRI			p=.310	N/S		p=.084
Isointense vs Hyperintense (ref.)	3.70 (0.71 to 19.20)					
Hypointense vs Hyperintense (ref.)	1.57 (0.62 to 3.96)					
Arachoid layer: Interrupted vs Intact (ref.)	1.06 (0.44 to 2.56)		p=.895	N/S		p=.186
Contrast enhancement: Heterogeneous vs Homogeneous (ref.)	2.82 (1.19 to 6.70)		p=.015	N/S		p=.084
Tumour shape: Irregular vs Regular (ref.)	3.33 (1.33 to 8.30)		p=.007	N/S		p=.121
Tumour calcifications: Present vs Absent (ref.)	0.87 (0.29 to 2.65)		p=.808	N/S		p=.827
Capsular contrast enhancement: Present vs Absent (ref.)	0.97 (0.37 to 2.58)		p=.372	N/S		p=.861

Adjusted for gender, PTBE volume was identified as the only independent predictor for brain invasive growth (Abbreviations: N/S: not specified because not selected in multivariable analysis, OR: Odds ratio, CI: Confidence interval, p: p-value of likelihood ratio / score test for selected / non-selected variables, ref.: reference group).

**Figure 2:**
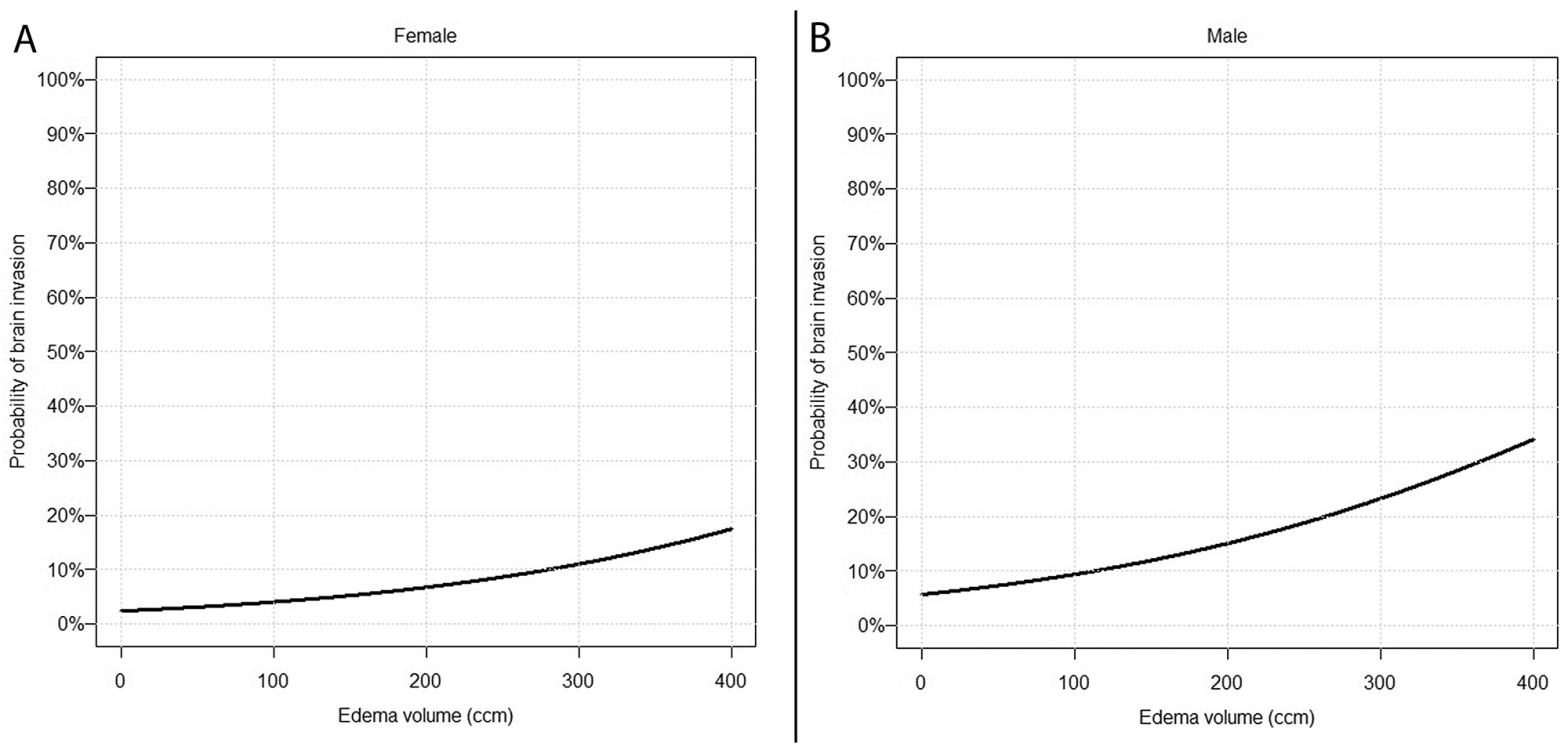
Prediction of brain invasion using findings on preoperative MRI Predicted probability of brain invasive growth depending on PTBE volume in females **(A)** and males **(B)** according to the final multivariable model (Table 2).

Multivariable analysis revealed edema volume (OR: 1.005 per ccm; 95% CI 1.002-1.009; p=.010) as the predominant predictor for brain invasive growth after adjustment for gender (OR (female=ref.): 2.45; 95% CI 0.81-7.40; p=.113). No interactions were found. The gender-specific probability for brain invasion as edema volume varies is visualised in Figure 2.

## DISCUSSION

The clinical and scientific importance of the detection of brain invasive growth in meningiomas has substantially increased with release of the 2016 edition of the WHO Classification of Central Nervous System Tumours [1]. Although the histopathological diagnosis is clearly defined, descriptions of the neuropathological assessment distinctly vary among the studies published so far [2]. On the other hand, incomplete tumour resections and the intraoperative utilization of Cavity Ultrasonic Surgical Aspirators (CUSA) can distinctly reduce tumour tissue available for histopathological analyses and therefore hinder the detection of brain invasion [5, 9]. Correspondingly, a broad range of frequency of brain invasion in meningiomas has been reported [2], further delineating the necessity to improve the sensitivity of the detection of brain invasion.

Only very few clinical risk factors associated with brain invasion are known to date and intraoperative assessment is insufficient [2]. A few studies revealed an increased rate of males, preoperative behaviour changes and seizures in patients harboring brain invasive as compared to non-invasive meningiomas [6, 7, 14–17]. Recently, we also demonstrated a distinctly increased risk of postoperative hemorrhage after surgery for brain invasive meningiomas (OR: 3.31, 95% CI 1.36-8.07; p=.009) [8]. Similarly, associations of radiological findings on preoperative imaging with brain invasive growth are sparsely investigated. Previous studies showed associations between MRI findings, such as indistinct meningioma/brain surface or heterogeneous contrast enhancement with high-grade histology in both adult and pediatric meningiomas [10, 12, 18, 19]. However, brain invasion has not been separately considered in these studies.

In the current series, the frequency of brain invasion was according to the broad range of previous reports [2]. Brain invasive growth was strongly associated with the presence of further histopathological high-grade criteria, thus indicating both the necessity of the inclusion of the latter in subsequent multivariable analyses and to identify radiological findings specifically predicting brain invasion.

As expected, high-grade histology was more common in tumours arising from the convexity and the falx [20]. However, in subgroup analyses, tumour location was only related with grading criteria other than brain invasion. Hence, although tumour location remains a strong predictor for high-grade histology, this finding cannot be attributed to the detection of brain invasion in these lesions.

In accordance with previous studies [12, 18, 19], heterogeneous contrast enhancement was correlated with high-grade histology. Similar findings were revealed when analyzing correlations of the heterogeneity of contrast enhancement with brain invasive growth or other grading criteria separately. Moreover, we identified an irregular tumour shape as a strong predictor for brain invasive growth but not for other histopathological high-grade criteria. In fact, chance of the detection of brain invasion on microscopic analyses was more than 3-fold higher in irregular compared to regular shaped meningiomas. Noteworthy, we showed that other variables characterizing the brain/tumour surface, such as contrast enhancement of the tumour capsule or disruption of the arachnoid layer, were insufficient to allow conclusions about brain invasive growth.

High-grade histology in our study was strongly correlated with increased tumour and edema volume. In accordance with previous studies, brain invasion was strongly associated with an increased PTBE volume [7, 11] in subgroup analyses. In contrast, increased PTBE volume was not related with other histological high-grade criteria (Figure 1). Vice versa, brain invasion was not associated with a larger tumour volume. Accordingly, multivariable analyses revealed only PTBE to be associated with brain invasion.

Although we identified several strongly associated MRI findings, none of these was found to be sufficient to exactly predict brain invasion alone. However, with Figure 2, we provide a simple and feasible tool, which helps to estimate the risk of brain invasion from routine preoperative radiological imaging. Consideration of these variables in communication between the neuroradiologist, the neurosurgeon and the neuropathologist might increase the sensitivity of the detection of brain invasion, e.g. by subjecting more tissue to histopathological analyses.

### Limitations of the study

The authors are aware of some limitations of the study. Basically, our study suffers the limitations of its retrospective nature. The low frequency of brain invasion required investigations in a large cohort and therefore a long inclusion period. On the other hand, preoperative MRI especially of patients who underwent surgery in the 90’s and early 2000’s was rarely available, which led to exclusion of a large portion of patients prior to any statistical analyses. Although we provide extensive and professional statistical analyses in a large patient collective, validation of our results in an external cohort is required to better evaluate the transferability and applicability during daily clinical routine. For technical reasons, 3D-volumetry could not be performed sufficiently but might have increased the accuracy of volume measurements. However, data from calculations did not significantly differ from those gained by volumetry in 20 representative cases (data not shown). While histopathological analyses and diagnosis were performed according to the current 2016 WHO classification of brain tumours, neuropathological analyses only included representative tissue samples but not the entire tumour.

## MATERIALS AND METHODS

### Clinical and histopathological data

Archives of the Institute of Neuropathology, Münster, Germany, were reviewed for all histo-pathologically confirmed primary diagnosed intracranial meningiomas resected in our neurosurgical department between 1991 and 2015. Clinical data were obtained from medical and operative reports as described previously [14, 21, 22] and included patients’ age at diagnosis, sex, the extent of resection according to the Simpson classification system [23] and preoperative Karnofsky Performance Score (KPS [24]).

Microscopic slices of all tumours were neuropathologically reviewed according to the current 2016 WHO criteria [1]. Correspondingly, brain invasion was analyzed on hematoxylin & eosin and Elastica van Gieson-stained slides and diagnosed in case of “irregular, tongue-like protrusions of tumour cells infiltrating underlying parenchyma, without an intervening layer of leptomeninges” (as illustrated in Figure 1 in reference [14]), and was considered as a stand-alone grading criterion for atypia. Further criteria of atypia or anaplasia were registered according to the WHO classification and are summarised as “other criteria” hereinafter.

### Radiological data

**Figure 3:**
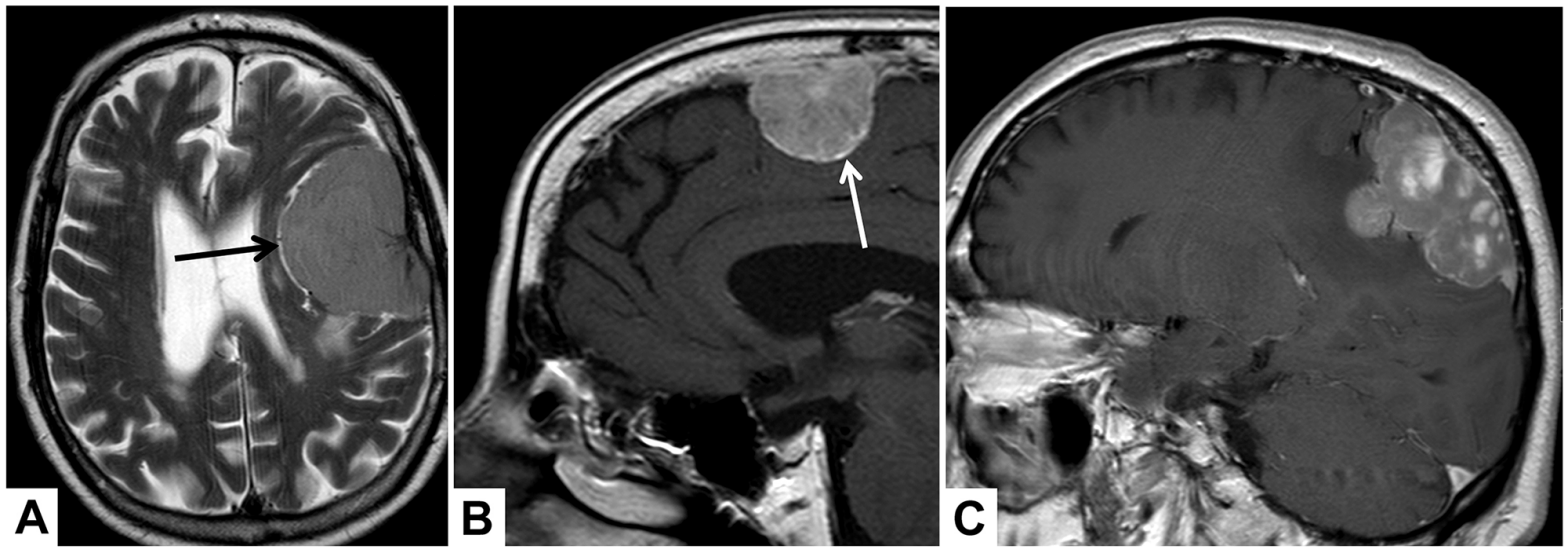
Illustrative examples of the analyzed MRI variables In **(A)**, axial T2-weighted MRI shows cerebrospinal fluid at the brain/meningioma border (arrow), indicating a distinct tumour surface with an intact arachnoid layer. In **(B and C)**, sagittal T1-weighted images show a contrast-enhancing tumour capsule (B, arrow), a heterogeneous gadolinium enhancement (C) and an irregular tumour shape with mushroom-like growth (C).

Patients were included in case of available sufficient preoperative MRI, defined as available axial T1 contrast-enhanced images. Preoperative MRI was analyzed by a team of two radiologists (PBS and AA) blinded to any histopathological data and disagreement was dissolved through discussion. Tumour location was dichotomously classified as “convexity or falx/parasagittal” and “other locations”. Tumour and edema volumes (V_T_ and V_E_) were estimated using the established formula for a spheroid V=4/3 × π × r1 × r2 × r3, where “r” is the tumour radius at the site of its largest extension in axial (r1), coronal (r2) and sagittal (r3) planes [7]. According to previous studies investigating assocations between MRI and WHO grade or patient’s prognosis, the following radiological variables were investigated (see illustrative MRI examples in Figure 3): Integrity of the arachnoid layer was analyzed on T2 imaging and was diagnosed as intact in case of a sharp tumour border and/or evidence of cerebrospinal fluid at the brain/meningioma surface [13]. Capsular enhancement and tumour shape were evaluated on gadolinium-enhanced T1-weighted imaging and dichotomously registered as absent/present and regular or irregular, respectively [12, 13]. Similarly, pattern of contrast enhancement was registered as heterogeneous or homogenous on T1-weighted images [13]. Intensity of the tumour and presence of intratumoural calcifications were analyzed on T2 images and classified as hyper-, iso- or hypointense as compared to the grey matter and present or absent, respectively [12].

Both tumour and edema volumes as well as brain invasion had been registered earlier for a previous study in a subset of patients [7, 14]. Data collection and scientific use were approved by the local ethics committee and permitted by the patient in each single case (Münster 2007-420-f-S and Münster 2018-061-f-S).

### Statistical analysis

Calculations were performed using standard commercial statistic software (IBM Corp. Released 2017. IBM SPSS Statistics for Windows, Version 25.0. Armonk, NY, USA: IBM Corp.). Data are described by absolute and relative frequencies for categorical and by median and range for continuous variables, respectively. Fisher’s exact and Mann-Whitney-U tests were performed to compare two independent samples regarding a categorical and continuous outcome, respectively.

Logistic regression was used to predict the risk of brain invasion, based on observed clinical and radiological data. Multivariable analysis was performed with forward stepwise selection (inclusion criterion: score test p-value ≤.05; exclusion criterion: likelihood ratio test p-value >.10) based on the variables summarised in Table 2 while adjusting for gender. Pairwise interactions were assessed in a second block. The results are summarised as odds ratios (OR) with 95%-confidence intervals (CI), and likelihood ratio test p-value for selected variables. For non-selected variables, the p-value of the score test is given. ROC analysis was performed to identify a cut-off point for edema volume as discrimination threshold for brain invasion. Maximality of Youden’s index was used as criterion for selecting the optimum cut-off point. All analyses were regarded as explorative. Therefore, no significance level was fixed. Reported p-values are two-sided and considered as descriptive measures to detect and study meaningful effects (with a cut-off at 5% for statistical noticeability).

## CONCLUSIONS

In conclusion, we found several MRI based markers that can serve to predict brain invasive growth, independent of further histopathological high-grade criteria. Moreover, we were able serve a tool referring to routine preoperative imaging, which helps to estimate the risk of brain in neuropathological analyses. Hence, our findings might lead to more focused histopathological analyses and can therefore improve the detection of brain invasion.
